# Spontaneous cerebrospinal fluid leak of the sphenoid sinus mimicking allergic rhinitis, and managed successfully by a ventriculoperitoneal shunt: a case report

**DOI:** 10.1186/s13256-016-1107-0

**Published:** 2016-11-03

**Authors:** Youssef Darouassi, Mohamed Mliha Touati, Mehdi Chihani, Ali Akhaddar, Haddou Ammar, Brahim Bouaity

**Affiliations:** 1 ENT Department, Military Hospital Avicenna, Marrakech, Morocco; 2 Neurosurgery Department, Military Hospital Avicenna, Marrakech, Morocco

**Keywords:** Spontaneous cerebrospinal fluid leak, Sphenoid sinus, Ventriculoperitoneal shunt, Case report

## Abstract

**Background:**

Spontaneous cerebrospinal fluid leaks are rare but may lead to confusion with other diseases in patients without history of trauma. We report a rare case unusual for two reasons. First, our patient was put under antiallergic medication for months before the diagnosis of spontaneous cerebrospinal fluid leak of the sphenoid sinus. Second, our patient was managed successfully by a ventriculoperitoneal shunt.

**Case presentation:**

Our patient was a nonobese 49-year-old Arab man without history of trauma or surgery who presented with rhinorrhea. He was given allergic rhinitis medication for 4 months without improvement. After the onset of headache leading to the suspicion of paranasal sinusitis, a computed tomography scan discovered an osteodural defect in the sphenoid sinus roof and a magnetic resonance imaging scan showed an aspect of empty sella with an arachnoidocele. An eye fundus examination found papilledema suggesting the diagnosis of idiopathic intracranial hypertension. We performed a ventriculoperitoneal shunt without repair of the osteodural defect. Because of the favorable evolution, we decided to postpone surgery.

**Conclusions:**

Spontaneous cerebrospinal fluid leak should be considered even in nonobese male patients without history of trauma. Our observation adds to other case reports suggesting the decrease of cerebrospinal fluid pressure alone as an option for the treatment of spontaneous cerebrospinal fluid leaks. Additional studies are necessary to clarify the indications.

## Background

Cerebrospinal fluid (CSF) leaks are rare, but their complications, such as meningitis or brain abscess, are life-threatening [1]. They result from an abnormal communication between the subarachnoid space and the extracranial space [2]. Galen was the first to report recurrent nasal discharge of CSF in 200 B.C. and the entity was thought to be physiological until Thomson reported in 1899 a series of 21 cases with spontaneous CSF rhinorrhea (CSFR) as an abnormal phenomenon [3–5]. The largest series of patients with CSFR consisted of 161 cases: 43 % were traumatic, 29 % were secondary to endonasal surgery, 22 % of tumoral cause, and less than 5 % were idiopathic [6].

We describe the case of a patient who presented with CSFR due to idiopathic intracranial hypertension (IIH) with a breach in the sphenoid sinus roof and an aspect of empty sella and arachnoidocele on magnetic resonance imaging (MRI). In the light of this observation and a review of the literature, we will discuss the different aspects of this rare entity.

## Case presentation

**Fig. 1:**
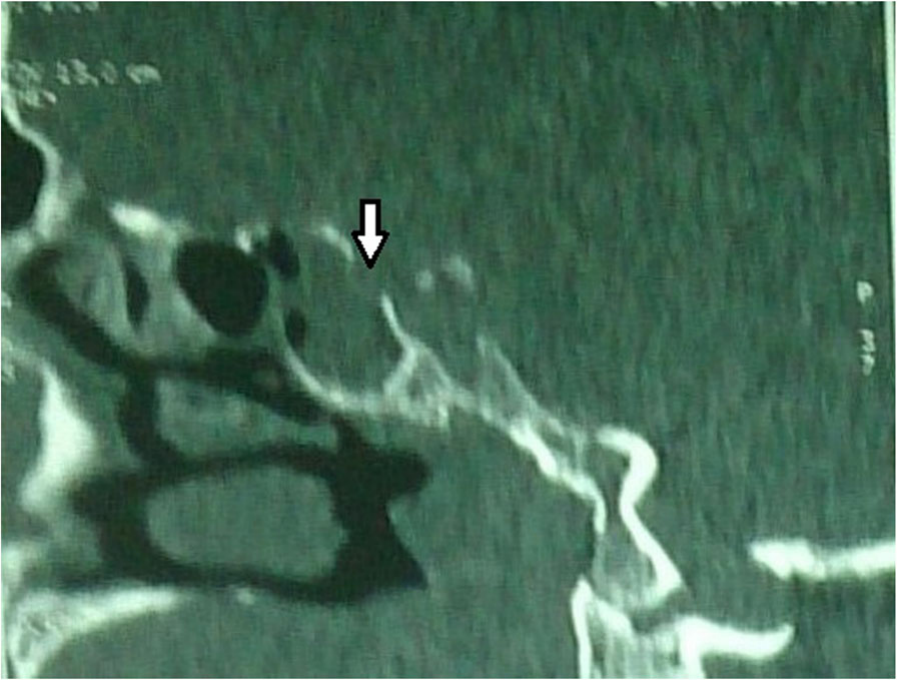
Computed tomography scan (sagittal cut) showing a filling of the sphenoid sinus with a breach (*arrow*) in the sinus roof

**Fig. 2:**
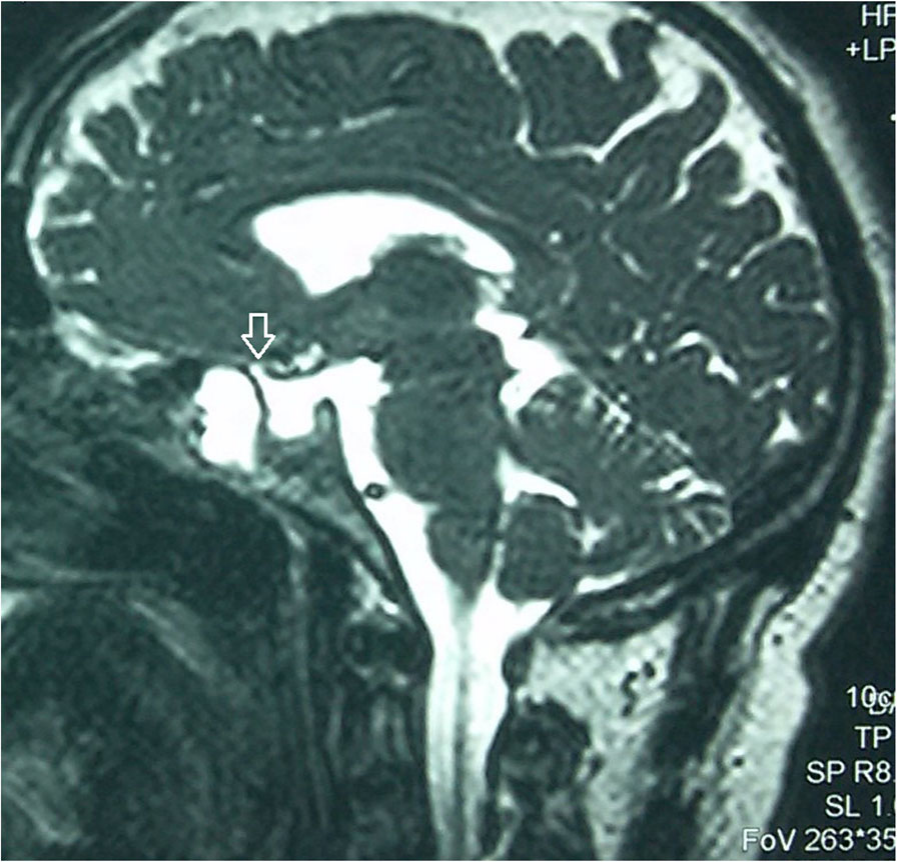
T2-weighted magnetic resonance imaging scan (sagittal cut) showing an empty sella associated with a pituitary arachnoidocele and fistula (*arrow*)

**Fig. 3:**
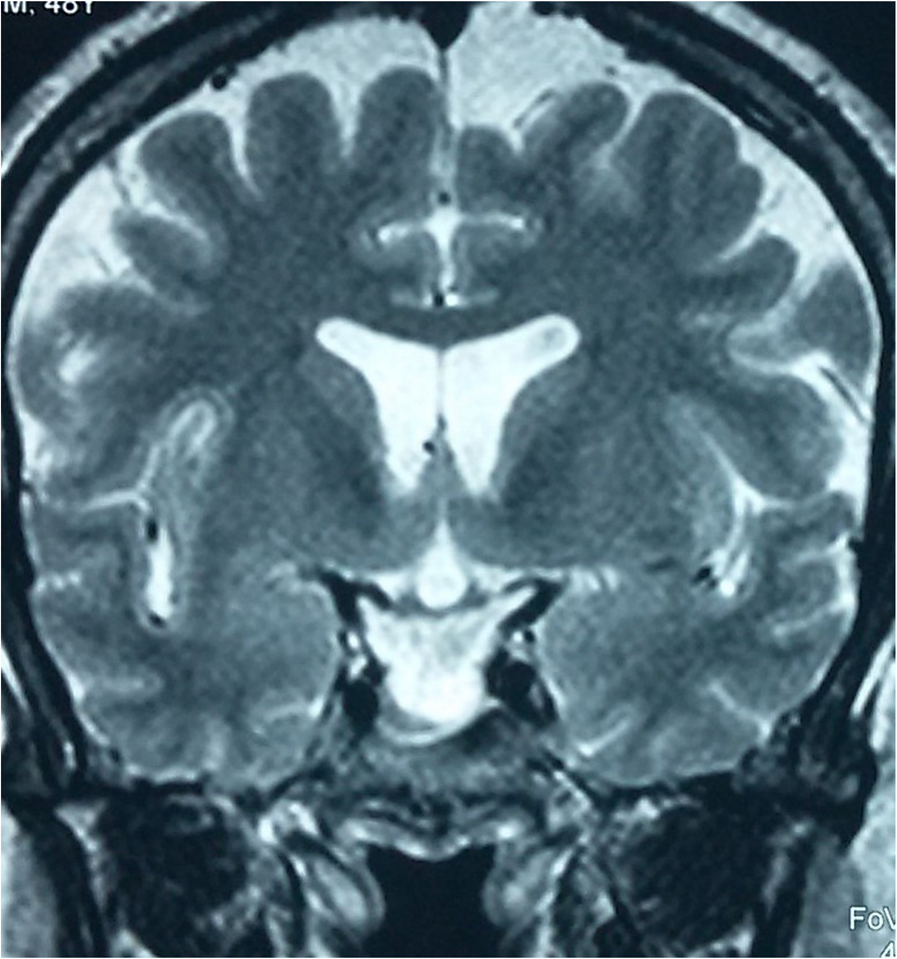
T2-weighted magnetic resonance imaging scan (coronal cut) showing the empty sella

We report the case of a 49-year-old Arab man with no history of head injury, obesity or high blood pressure. The onset of his symptoms was about 4 months earlier with a flu-like syndrome followed by a persistent clear bilateral rhinorrhea. The diagnosis of allergic rhinitis was suspected and our patient was given antihistamine and nasal corticosteroids without improvement. He presented to our outpatient clinic with a slight headache without fever. His overall health was maintained and a neurological examination was normal. A computed tomography (CT) scan showed a hypertrophy of the inferior nasal turbinates and a soft tissue density filling in the right sphenoid sinus with thinning and attenuation of its roof and a posterior bony defect at this level (Fig. 1). An MRI scan discovered an empty sella associated with a pituitary arachnoidocele (Figs. 2 and 3).

An eye fundus examination discovered signs of intracranial hypertension with early papilledema without functional impairment. Hormonal determination found normal thyroid-stimulating hormone (TSH), free T3 and T4, cortisol, adrenocorticotropic hormone (ACTH), lactate dehydrogenase (LDH), follicle-stimulating hormone (FSH) and luteinizing hormone (LH) with a slight prolactin elevation to 369.5 μUI/mL (normal 86 to 324 μUI/mL).

Our patient was given acetazolamide, ranitidine, paracetamol and amoxicillin with clavulanate. A ventriculoperitoneal shunting was performed resulting in an improvement of the rhinorrhea. The evolution was favorable with disappearance of the rhinorrhea in the last follow-up carried out 1 year after the procedure. The postoperative ophthalmological evaluation including a fundus examination was normal. Due to the favorable evolution, we decided to postpone surgery.

## Discussion

The term “spontaneous” CSFR has been applied to the cases unrelated to trauma, surgery, malformation, tumor, or previous radiation therapy [5, 7, 8]. The percentage among all causes of CSFR ranges from 6 to 23 % [5, 7, 9]. All CSF leaks have a common pathophysiology: disorder of arachnoid, dura and bone, and a CSF pressure that is either continuously or intermittently greater than the healing tensile strength of disrupted tissues [9, 10]. There is great evidence that the principal cause of the vast majority of spontaneous CSF leaks is elevated intracranial pressure (ICP), and that the entity represents a variant of IIH, also called pseudotumor cerebri [1, 10, 11].

Patients presenting CSFR without history of surgery or trauma are classically obese, hypertensive, multiparous women in their fourth or fifth decades, with women outnumbering men 2:1 [12]. The clinical manifestation is usually the same including rhinorrhea and, in some cases, otorrhea [10]. Patients often present with pressure-like headaches and pulsatile tinnitus [11]. On the other hand, patients with IIH are typically obese females who may present with headache, pulsatile tinnitus, and visual disturbances [1]. However, our patient was a 49-year-old man without history of trauma or obesity. This clinical presentation with a flu-like syndrome led to the misdiagnosis of allergic rhinitis and our patient was given antiallergic medication for months. After the appearance of headache, paranasal sinusitis was suspected, and a CT scan finally permitted us to make the correct diagnosis.

The classical site of the breach is located in the anterior cranial fossa [7, 13]. However, the most common location has varied in the literature, and the cribriform plate and the lateral recess of the sphenoid sinus became the most frequently quoted areas [1]. Fistulas in the sphenoid sinus are usually located at the junction between the anterior portion of the lateral wall of the sinus and the floor of the base of the skull (82 %), or at the middle portion of the sidewall of the sinus (18 %) [13]. The location of Sternberg’s canal in anatomic studies is inconsistent with the majority of lateral sphenoid sinus CSF leaks; thus, a congenital etiology underlying lateral recess of the sphenoid sinus CSF leaks has been largely disproven, and mention of this should be removed from the literature [1]. Extensive pneumatization of the sphenoid sinus (pneumosinus dilatans) can play a role in the pathogenesis [1, 7, 9, 10]. Fistulas in the posterior wall of the sphenoid sinus are very rare [13]. Multiple defects exist in up to 31 % of cases [1]. Our patient has a breach in the roof of the sphenoid sinus.

The localization of the breach is a neuroimaging challenge. It allows for the planning of the surgical approach, and requires a CT scan that should ideally be spiral with multiplanar reconstruction. The objective is to visualize the sinus fillings and the bone defect. However, MRI is a better choice because of its multiplanar acquisitions and nonradiating character [7]. Advances in CT and MRI techniques have improved sensitivity, which amounted to 88–93 % for high-resolution CT and to 89–100 % for MRI cisternography even in patients with inactive leaks. Therefore, the two techniques or a combination of them has replaced the previously used invasive procedures [5].

The MRI aspect of empty sella is highly associated with spontaneous CSF leaks and IIH [1]. A study proved its reversibility after control of elevated ICP [14]. High pressure is exerted on sites of weakness, including the fascia of the sellar diaphragm, with herniation of the meninges through the sellar diaphragm resulting in the appearance of the empty sella on MRI [11]. Our case illustrates this situation perfectly with an empty sella on MRI, and early papilledema discovered at the eye fundus examination; due to elevated ICP.

The content of osteodural defects is variable: meningocele, meningoencephalocele, encephalocele, meningeal or arachnoid hernia, arachnoid diverticulum, or arachnoid cyst. This content may modify the preoperative planning and the grafting technique [5].

Acetazolamide, which was used in our case, is a diuretic of the carbonic anhydrase inhibitor type. It can decrease CSF production up to 48 % [15].

CSF diversion can be performed by lumboperitoneal or ventriculoperitoneal shunts, and occasionally by ventriculojugular and ventriculoatrial shunting. Complications of CSF diversion include shunt blockage, infection, abdominal and back pain, intracranial hypotension, and tonsillar herniation. No prospective controlled trials have been made to guide the choice of procedure [16]. Although, there is a growing body of evidence in favor of the use of ventriculoperitoneal shunts [17, 18], which is why we chose this procedure.

A subcranial approach may offer a broad exposure of the fronto-sphenoid-ethmoidal anterior midline and the posterior planes of the anterior fossa skull base, providing a safe and effective technique for repair of CSFR with the advantages of avoiding frontal lobe retraction [19]. However, the endoscopic technique became the primary approach to the treatment of CSF leaks due to its many advantages such as safety and fast postoperative recovery without facial scars [20]. Wigand first reported a successful procedure in 1981 [21]. The success rate of the endoscopic technique is reported as 90 % at the first attempt, increasing to 97 % at the second attempt [2, 7, 20]. The defects can be covered by materials including a tissue graft such as abdominal fat, nasal mucosa, galeal frontalis flap, musculature, lyophilized dura mater or fascia lata, which is very suitable because it is autologous material and does not shrink in the way that other tissues, such as muscle, do [19, 22]. Repair of the breach may be also accomplished with bone cement (hydroxyapatite) [22]. Even if spontaneous CSF leaks have the highest recurrence rate, recent studies support the role of decreasing ICP via medication, weight loss, or CSF diversion to improve outcome after endoscopic repair [1, 11, 23].

Of all the CSF leaks, those from the sphenoid sinus represent a unique challenge due to the anatomical rapport and the extreme variability in the shape of the sinus [2]. In our case, a ventriculoperitoneal shunt was performed resulting in the cessation of the rhinorrhea and the disappearance of the papilledema. Given the favorable evolution, and the location of the breach, we decided to postpone surgery. The presence of a foreign body should not be problematic with a close follow-up and since shunt surgery has, for example, been an established and widely accepted treatment for congenital hydrocephalus. Our observation adds to other case reports suggesting a decrease of CSF pressure alone for the treatment of some cases of CSFR. Ransom reported, for example, the case of a patient with IIH who presented a spontaneous CSF leak after the failure of ventriculoperitoneal shunting. Cessation of the CSFR was observed at 1-year follow-up after revision of the shunt [22].

## Conclusions

There is overwhelming evidence in the literature that the underlying cause of the vast majority of spontaneous CSF leaks is elevated ICP, and that the entity represents a variant of IIH [1, 10, 11]. The clinical presentation is various and may lead to confusion like in our case. The assessment is based on imaging techniques. A decrease of CSF pressure alone for the treatment could be considered in some cases as illustrated by our observation, where a ventriculoperitoneal shunting was performed with good results. However, further studies are necessary.

## Abbreviations

CSF: cerebrospinal fluid
CSFR: cerebrospinal fluid rhinorrhea
CT: computed tomography
ICP: intracranial pressure
IIH: idiopathic intracranial hypertension
MRI: magnetic resonance imaging
